# The extricable links between health, wealth, and profits

**DOI:** 10.1136/bmj.k4418

**Published:** 2018-10-22

**Authors:** Gavin Yamey, Devi Sridhar, Kamran Abbasi

**Affiliations:** 1 Duke Global Health Institute, Duke University, Durham, NC, USA; 2 Medical School, Edinburgh University, Edinburgh, UK; 3 *The BMJ*, London, UK

## Abstract

A new BMJ series will examine the latest evidence

We live in critical times for global health. Big gains made during the millennium development goals era, including a halving of child mortality from 1990 to 2015,^1^ fuelled optimism about unabated—or even accelerated—progress. We hear talk of the end of AIDS,^2^ universal health coverage by 2030,^3^ and a pandemic-free world.^4^ But in the current political climate these outcomes are a distant dream. Our era is one of retrenchment and disinvestment in global health, throwing cold water on utopian rhetoric and threatening to reverse recent gains.

After health aid tripled between 2000 and 2010 from around $10bn (£7.6bn; €8.7bn)to $30bn annually, such investments are now stagnating “amid political uncertainty, expanding and ag[e]ing populations, and emerging pandemics.”^5^ The HIV pandemic is not on track to end,^6^ yet last year eight out of 14 bilateral donors cut their support for HIV programmes.^7^

The only feasible way to reach universal health coverage and other health related sustainable development goals is through mobilisation of domestic resources by low and middle income countries. But this remains elusive. Indeed, the trend in low income countries is in the wrong direction: the World Health Organization found that since 2000 these countries have reduced their allocation of domestic resources to health.^8^ Health is being deprioritised in national budgets worldwide, not just in low income countries. Health and social spending in the United Kingdom, for example, has slowed down in recent years, a plausible explanation for several deteriorating health outcomes.^9^

A paradox lies at the heart of this retreat from health. It comes at a time when the evidence on the benefits of increasing donor and domestic health investments is at its strongest. In response, *The BMJ* is launching a new series to capture this evidence and restate the profound social and economic benefits of investing in health.

The launch is timed with two events that can help restore health to the top of the global development agenda. Firstly, the global conference on primary healthcare in Astana, Kazhakstan, intends to reinvigorate global commitments to universal health coverage. It will mark the 40th anniversary of the Alma Ata declaration, which launched the global primary healthcare movement.^10^ Secondly, the World Bank has just released a new index that ranks countries on their human capital, defined by the bank’s president as “the sum total of a population’s health, skills, knowledge, experience, and habits.”^11^ The rankings primarily reflect national investments in health and education.

*The* *BMJ* series on health, wealth, and profits has three themes. The first is the link between health and wealth, documented by 2001’s influential Commission on Macroeconomics and Health.^12^ Since then, several avenues of research using newer economic approaches show that the returns on investing in health are even greater than previously believed.^13^

But where can the greatest gains be made? Non-communicable diseases, which encompass cardiovascular diseases, diabetes, cancer, and chronic respiratory diseases, are responsible for 41 million deaths a year (71% of all deaths globally), of which 15 million are in people aged 30 to 69.^14^ Over 85% of these early deaths occur in low and middle income countries.^14^

Thus, the second theme in our series will be the rising cost of non-communicable diseases to societies, and the losses in both health and wealth to households, health systems, and national economies. Four modifiable risk factors drive the non-communicable disease epidemic: tobacco consumption, alcohol misuse, poor diet, and physical inactivity.^14^ The first three of these are heavily influenced by the marketing, sponsorship, and promotion strategies of large multinational corporations such as Philip Morris, Heineken, and Nestlé.^15^

How then can governments, citizens, and societies begin to make progress on regulating large private stakeholders in global health? International collective action, global regulatory frameworks, and other efforts to tackle key risk factors for poor health form the third theme of our series. As tobacco control is most often pointed to as a success, we will analyse lessons from the Framework Convention on Tobacco Control and consider where new instruments and approaches are necessary.

Our broad vision for this series is to reignite the debate on investing in health and healthcare systems, with a focus on non-communicable diseases. Policy makers can no longer point to a lack of evidence on the links between health and wealth, ill health and losses to the economy, and the influence of commercial determinants of health. We invite contributions to this series that are relevant to our three themes. As the commissioned papers in this series will show, a solid evidence base drawn from public health, epidemiology, economics, and political science already exists on how to move forward and create a world focused on healthier lives instead of corporate profits.

## Notes

Provenance and peer review: Commissioned; not externally peer reviewed.
